# Association between serum potassium levels and haematoma expansion in intracerebral hemorrhage: a retrospective cohort study

**DOI:** 10.3389/fneur.2026.1707430

**Published:** 2026-03-18

**Authors:** Rong Wu, Min Jiang, Bing Bao, Qi Li, Jiaojiao Meng, Meili Shen, Jian Wang, Xiaoping Yin, Moxin Wu

**Affiliations:** 1 Department of Medical Laboratory, Affiliated Hospital of Jiujiang University, Jiujiang, China; 2 Jiujiang Clinical Precision Medicine Research Center, Jiujiang, China; 3 Department of Neurology, Affiliated Hospital of Jiujiang University, Jiujiang, China; 4 Department of Neurology, The Second Affiliated Hospital of Anhui Medical University, Hefei, China; 5 Department of Human Anatomy, School of Basic Medical Sciences, Zhengzhou University, Zhengzhou, Henan, China; 6 Nanozyme Laboratory in Zhongyuan, Zhengzhou University, Zhengzhou, Henan, China; 7 State Key Laboratory of Antiviral Drugs, Pingyuan Laboratory, Zhengzhou University, Zhengzhou, Henan, China

**Keywords:** cerebral hemorrhage, haematoma expansion, neuroscience, potassium, stroke

## Abstract

**Objective:**

Serum potassium levels are risk factors for intracerebral hemorrhage (ICH), and haematoma expansion (HE) is an important determinant of poor prognosis in patients with ICH. This study investigated the correlation between serum potassium levels and HE after ICH.

**Materials and methods:**

This retrospective study analyzed serum potassium levels in ICH patients. On the basis of imaging criteria (haematoma volume increase ≥33% or absolute enlargement >6 ml), patients were categorized into the HE subgroup. Differences in serum potassium levels were compared using the Mann–Whitney *U*-test. Propensity score matching (PSM) was applied to balance baseline characteristics between the HE subgroup and the non-HE subgroup for further comparison. Additionally, adjusted logistic regression analyses and receiver operating characteristic (ROC) curves were employed to evaluate the correlation between HE and serum potassium levels.

**Results:**

A total of 310 patients with ICH were identified, of whom 50 (16.1%) had HE. After PSM, patients in the HE subgroup presented lower potassium levels than non-HE patients did (3.71 ± 0.52 vs. 3.92 ± 0.52, *p* = 0.009). Low serum potassium levels were significantly associated with HE (adjusted Odds Ratio (aOR) = 0.36; 95% CI: 0.15–0.80; *p* = 0.017), with an AUC value of 0.635.

**Conclusion:**

Low serum baseline potassium levels are associated with a higher risk of HE after ICH. The relevance of serum potassium levels to severity after ICH is emphasized.

## Introduction

Intracerebral hemorrhage (ICH), a subtype of stroke caused by cerebrovascular rupture, accounts for approximately 3.28 million new cases annually worldwide. This represents approximately 28.8% of all stroke types and is associated with high rates of disability and mortality (1, 2). Haematoma expansion (HE), which occurs in approximately 30% of ICH patients within the first 24 h, is a major determinant of early neurological deterioration and poor clinical outcomes (3–5). Neuroimaging, particularly computed tomography (CT), remains the gold standard for diagnosing ICH and assessing haematoma status (6). The “spot sign” observed on CT angiography is a significant imaging marker (7) for predicting HE and facilitates the identification of patients who may require urgent intervention. Recent studies suggest that biochemical markers, such as serum electrolyte levels, may also provide additional prognostic information (8–12).

Electrolyte imbalances are frequently observed in acute stroke and may reflect underlying pathophysiological responses to brain injury. In particular, the acute neuroendocrine stress response to ICH—mediated by activation of the sympathoadrenal axis and hypothalamic-pituitary-adrenal axis—leads to increases in catecholamines and cortisol. These hormonal changes can drive intracellular potassium shifts, resulting in reduced serum potassium concentrations (13, 14). Clinical studies have reported significantly lower serum potassium levels in patients with ICH than in those with ischaemic stroke (15).

Experimental models have also implicated potassium homeostasis in the pathogenesis of ICH. In a collagenase-induced ICH mouse model, deletion of the TREK-1 potassium channel was shown to impair vascular endothelial integrity and disrupt the blood–brain barrier, potentially exacerbating haemorrhagic progression (16). Despite these mechanistic insights, the association between serum potassium levels at admission and HE in ICH patients remains unclear.

This study aimed to investigate the relationship between baseline serum potassium levels and HE in patients with spontaneous ICH, with the goal of identifying its potential as a biomarker.

## Methods

### Study cohort

This real-world retrospective cohort study was conducted between January 31, 2020 and December 31, 2024 in patients with acute spontaneous ICH diagnosed by head CT scans and admitted to Afffliated Hospital of Jiujiang University. All patients were hospitalized within 24 h after stroke, and their haematomas received non-operative treatment. The exclusion criteria were: (1) age less than 18 years; (2) surgical treatment; (3) ICH resulting from TBI, haemorrhagic transformation of cerebral infarction, intracranial aneurysm, intracranial tumors, arteriovenous malformation, venous sinus thrombosis, or moyamoya disease; and (4) other specific conditions, such as severe infections within the past month, known malignancies, and autoimmune diseases. All patients received standardized blood pressure management according to the AHA/ASA guidelines (17). This study was approved by the Institutional Review Boards at the Affiliated Hospital of Jiujiang University. Patient informed consent was waived in accordance with national legislation and institutional requirements.

### Data collection

The diagnosis and location of the ICH were verified by clinical neuroradiologists or trained study staff. All CT scans were performed without intravenous contrast injection. All CT images were saved in Digital Imaging and Communications in Medicine format and further reviewed independently by two experienced readers (B.B. and X.P.Y.) who were blinded to the clinical outcomes. Semiautomated CT volumetric measurements were performed using ITK-SNAP software (University of Pennsylvania, Philadelphia, USA; URL: http://www.itksnap.org) to assess haematoma volume. HE was defined as a haematoma growth of more than 33% and/or more than 6 ml in 24 h compared with the baseline haematoma. Peripheral venous blood samples were collected within 24 h of patient admission. Demographic data (age, sex), vascular risk factors (hypertension, diabetes mellitus), lifestyle factors (smoking, alcohol consumption), clinical and imaging parameters (systolic blood pressure on admission, baseline haematoma volume), and laboratory parameters (white blood cell count, blood glucose) were recorded.

### Serum potassium level detection

Baseline serum potassium was measured immediately upon admission (prior to intervention). Subsequent measurements were taken on Days 1, 3, 7, and 14. To measure serum potassium levels, blood samples were centrifuged at 3,000 rpm for 5 min, after which serum samples were separated and serum potassium levels were measured using a Beckman Coulter AU5831 automated biochemistry analyser (Beckman Coulter Inc., Brea, CA, USA) according to the manufacturer's instructions for the use of the Beckman Coulter Bioanalytical System buffer. Measurements were performed by senior laboratory technicians who were blinded to the clinical data to ensure objective and reliable results. The normal reference range for serum potassium in this laboratory is 3.5–5.5 mmol/L.

### Statistical analysis

Continuous variables were analyzed using the Mann–Whitney *U*-test, and categorical variables were compared with the chi–square test. To control for baseline confounders, we performed 1:3 propensity score matching (PSM) with the nearest neighbor method, setting a caliper width of 0.1. We evaluated predictive performance by plotting the receiver operating characteristic (ROC) curve and used a multivariable logistic regression model to assess the effect of serum potassium levels on HE. To evaluate the robustness of the findings, multiple imputation was used to assess the sensitivity to missing data. Influential points were identified based on Cook's distance, with the threshold set at 4/(n–p). The variance inflation factor (VIF) was calculated to diagnose multicollinearity. Internal validation was performed using the bootstrap method with 1,000 repetitions. The assessment of model calibration was performed via the Hosmer–Lemeshow test with 10 groups. The decision curve analysis (DCA) was performed to examine the clinical utility of the model. Statistical analyses were performed using R version 4.4.3 (MatchIt package version 4.7.2; pROC package version 1.18.5; mice package version 3.19.0; rmda package version 1.6; car package version 3.1-3; boot package version 1.3-31; rms package version 8.1-0; ResourceSelection package version 0.3-6). *p* < 0.05 was considered to indicate statistical significance.

## Results

### Patient selection and characteristics

**Figure 1:**
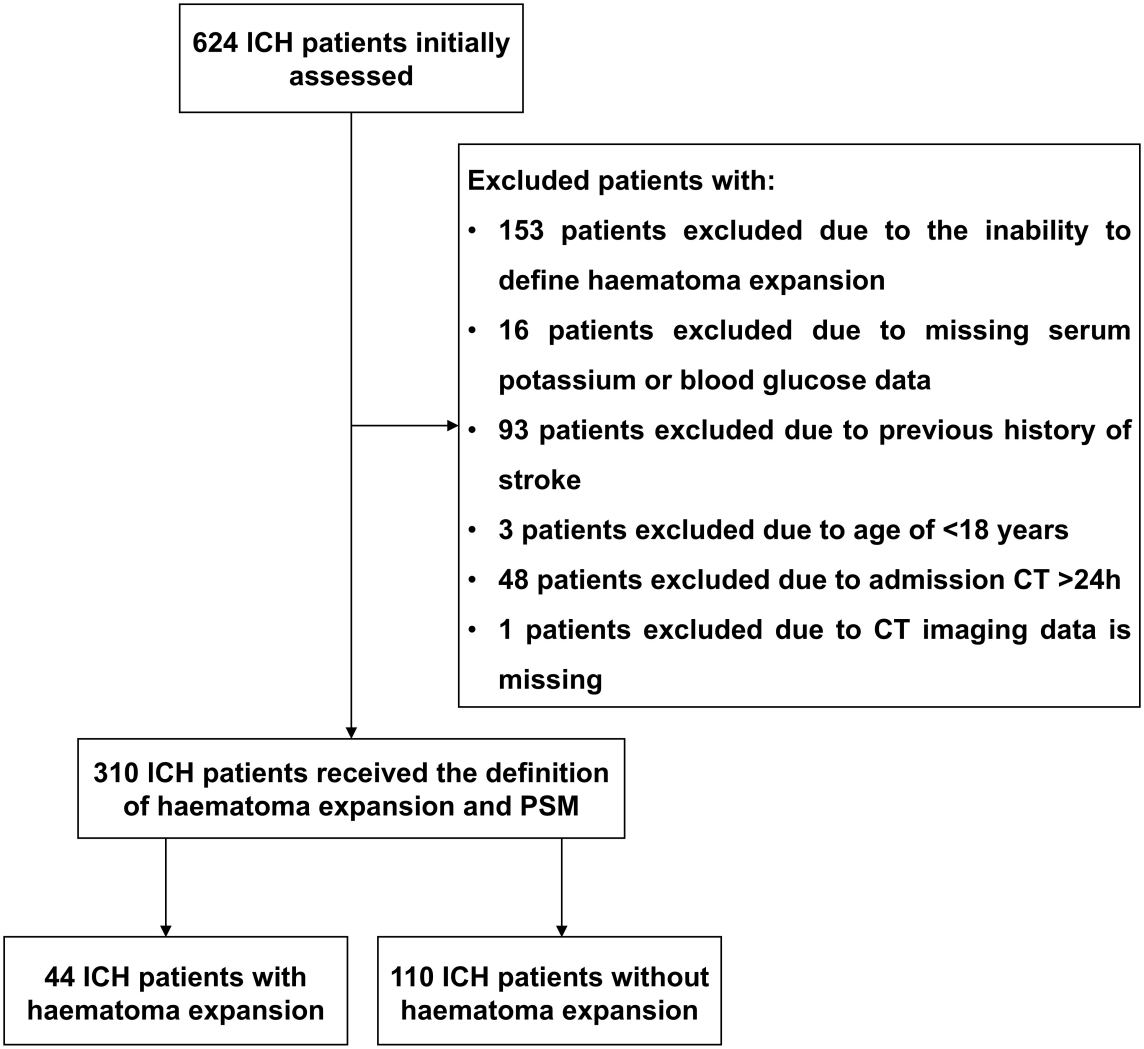
Flowchart of screening of eligible acute spontaneous ICH patients. Initially, we evaluated 624 patients with ICH and excluded 314 patients with ICH. After PSM, 154 patients with ICH were ultimately included, 44 in the HE subgroup and 110 in the non-HE subgroup. ICH, intracerebral hemorrhage; HE, haematoma expansion; PSM, propensity score-matched.

During the study period, a total of 624 patients with acute spontaneous ICH who were hospitalized within 24 h of symptom onset were initially included. Afterwards, 314 patients were excluded on the basis of the exclusion criteria shown in Figure 1. Finally, 310 patients with ICH were included in this study. Among them, 50 (16.12%) patients were in the HE subgroup. After PSM, 44 and 110 patients were in the HE and non-HE subgroups, respectively. Patient characteristics were well matched (Table 1). The demographic and clinical characteristics of the patients are shown in Table 1.

**Table 1:** Characteristics of ICH patients in total and PSM cohorts.

**Characteristic**	**Unmatched cohorts**			**PSM cohorts**			
	**Non-haematoma expansion (*****n*** = **260)**	**Haematoma expansion (*****n*** = **50)**	***p*** **-value**	**Non-haematoma expansion (*****n*** = **110)**	**Haematoma expansion (*****n*** = **44)**	**SMD**	***p*** **-value**
Male, *n* (%)	176 (67.69%)	38 (76.00%)	0.318	81 (73.63%)	33 (75.00%)	0.01	1.000
Age, years (mean ± SD)	63.29 ± 13.87	65.68 ± 13.92	0.267	65.04 ± 13.26	64.91 ± 14.28	0.1	0.971
Baseline haematoma volume	14,878.29 ± 16,180.68 (9,480.00 [15,585.75])	27,426.36 ± 26,109.17 (20,881.50 [26,959.25])	0.001^**^	17,579.55 ± 15,678.80 (14,767.50 [18,081.50])	21,347.30 ± 16,310.14 (16,559.00 [24,234.0])	0.01	0.112
Hypertension, *n* (%)	158 (60.76%)	33 (66.00%)	0.590	77 (70.00%)	29 (65.91%)	0.06	0.762
Diabetes mellitus, *n* (%)	25 (9.61%)	8 (16.00%)	0.275	14 (12.73%)	5 (11.36%)	0.02	1.000
Smoking, *n* (%)	61 (23.46%)	13 (26.00%)	0.837	27 (24.55%)	11 (25.00%)	0.05	1.000
Alcohol, *n* (%)	45 (17.30%)	11 (22.00%)	0.555	24 (21.82%)	9 (20.45%)	0.08	1.000
Admission systolic blood pressure	170.40 ± 27.62	175.02 ± 27.69	0.283	173.30 ± 29.28	175.16 ± 28.79	0.02	0.658
Blood glucose level (mmol/L)	8.19 ± 2.73	7.94 ± 2.41	0.643	7.73 ± 2.17	7.68 ± 2.16	0.03	0.815
Leucocyte count ( × 10 (9)/L)	8.95 ± 3.60	8.44 ± 3.29	0.411	8.87 ± 3.37	8.26 ± 3.36	0.1	0.282
Serum potassium level (mmol/L)	3.88 ± 0.49	3.71 ± 0.51	0.015^*^	3.92 ± 0.52	3.71 ± 0.52	–	0.009^**^

Quantitative data was expressed as mean ± standard deviation, and qualitative data were expressed as counts (proportions), median increase in baseline haematoma volume [IQR].

To compare different variables between groups, the chi–square test was used for qualitative data and the Mann–Whitney U-test for quantitative data.

ICH, intracerebral hemorrhage; PSM, propensity score-matched.

^*^p < 0.05.

### Correlation of low serum potassium levels with haematoma expansion

**Figure 2:**
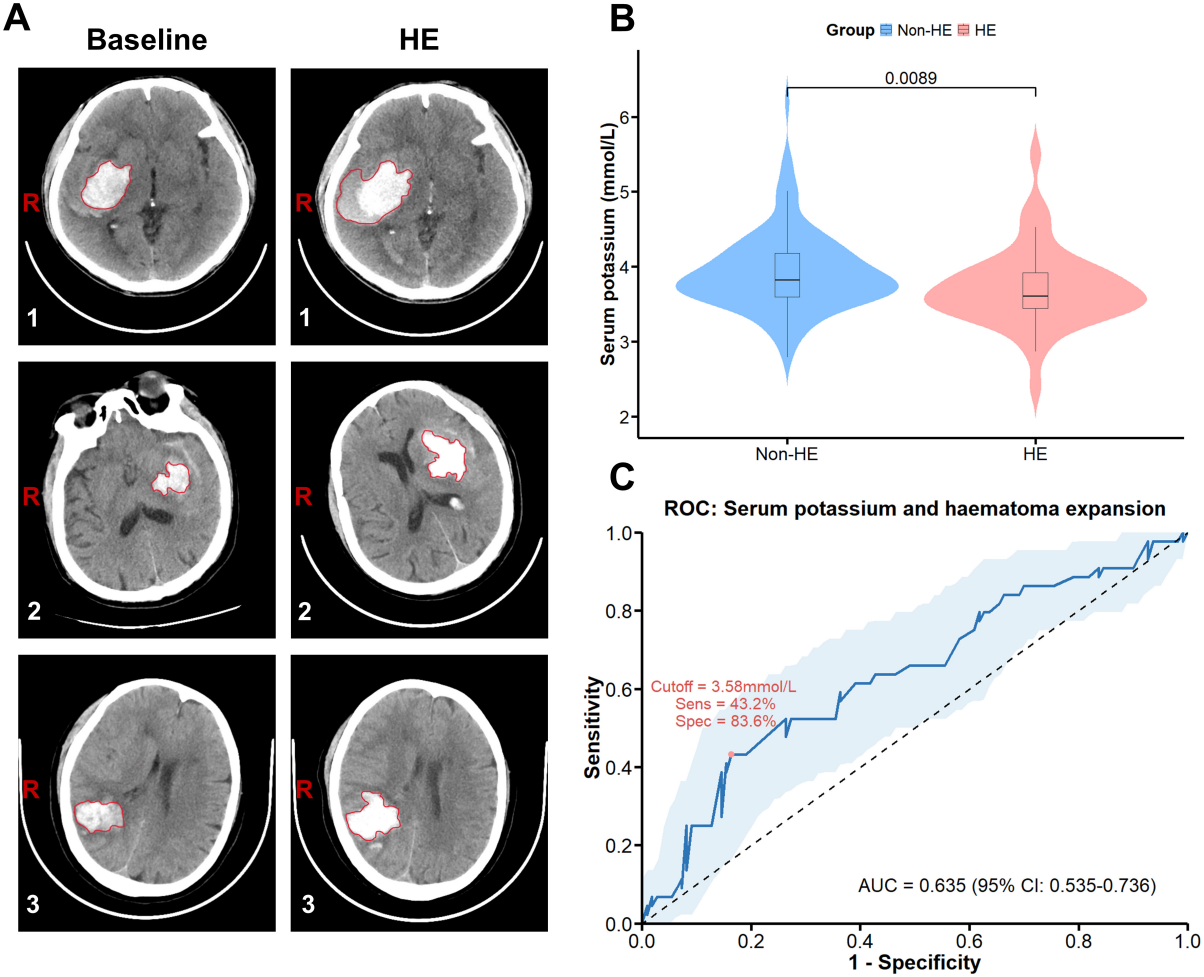
In the PSM cohort, low potassium levels were associated with HE in patients with ICH. **(A)** CT images of patients with ICH were used for analysis, with the left image representing a baseline haematoma and the right image representing HE. **(B)** Serum potassium levels in the HE subgroup and the non-HE subgroup. **(C)** ROC curve of serum potassium levels for HE. ICH, intracerebral hemorrhage; HE, haematoma expansion; ROC, receiver operating characteristic; AUC, area under the curve.

Figure 2A shows the representative CT imaging diagnoses of the patients used for analysis. In the unmatched cohort, serum baseline potassium levels were lower in the HE subgroup than in the non-HE subgroup (3.71 ± 0.51 vs. 3.88 ± 0.49, *p* = 0.015) (Supplementary Figure S1). After PSM, serum baseline potassium levels were significantly lower in the HE subgroup than in the non-HE subgroup (3.71 ± 0.52 vs. 3.92 ± 0.52, *p* = 0.009; Figure 2B). Moreover, after adjusting for baseline confounders by multifactorial logistic regression (Table 2), low serum potassium levels were significantly associated with HE (adjusted Odds Ratio (aOR) = 0.36, 95% CI: 0.15–0.80; *p* = 0.017). To assess its discriminative ability, the ROC curve for serum potassium levels and HE yielded an area under the curve (AUC) of 0.635 (95% CI: 0.535–0.736) (Figure 2C).

**Table 2:** Univariate and multivariate logistic regression analysis for HE in the PSM cohort.

**Clinical characteristics**	**Univariable analysis**		**Multivariable analysis**	
	**OR (95% CI)**	***p*** **-value**	**OR (95% CI)**	***p*** **-value**
Age	0.99 (0.97–1.02)	0.955	1.00 (0.97–1.03)	0.892
Gender	1.07 (0.49–2.47)	0.861	1.23 (0.51–3.08)	0.651
Baseline haematoma volume	1.00 (0.99–1.00)	0.186	1.00 (0.99–1.00)	0.133
Hypertension	0.83 (0.40–1.72)	0.621	0.85 (0.37–1.99)	0.701
Diabetes mellitus	0.88 (0.27–2.47)	0.816	1.47 (0.37–5.34)	0.566
Smoking	1.02 (0.44–2.26)	0.953	0.86 (0.30–2.37)	0.779
Alcohol	0.92 (0.37–2.12)	0.852	1.05 (0.35 −3.05)	0.923
Admission systolic blood pressure	1.00 (0.99–1.01)	0.720	1.00 (0.99–1.01)	0.798
Blood glucose level	0.99 (0.83–1.16)	0.880	0.96 (0.76–1.19)	0.696
Leucocyte count	0.94 (0.83–1.05)	0.312	0.92 (0.79–1.04)	0.212
Serum potassium level	0.41 (0.18–0.87)	0.027^*^	0.36 (0.15–0.80)	0.017^*^

ICH, intracerebral hemorrhage; PSM, propensity score-matched; HE, haematoma expansion.

^*^p < 0.05.

In the validation analysis (Supplementary Figure S2), stability analysis supported the reliability of the results, while the model itself demonstrated good calibration (Hosmer–Lemeshow test, *p* = 0.27). DCA confirmed its strong clinical utility. In addition, we employed 1:1 nearest neighbor matching to obtain complete serum potassium level data for Days 1, 3, 7, and 14 from the HE subgroup (*n* = 28) and non-HE subgroup (*n* = 32). On this basis, we analyzed changes in serum potassium levels between the two subgroups at the aforementioned time points. (Supplementary Table S1). The results revealed that serum potassium levels significantly changed in the HE subgroup during each corresponding period, especially during the D7–D14 period (*p* = 0.0083). The connecting lines of individual serum potassium values had a large degree of dispersion and a high fluctuation range, indicating that the magnitude of change in serum potassium levels in the HE subgroup was significantly greater than that in the non-HE subgroup (Supplementary Figure S3). This model demonstrates modest predictive performance; its primary value lies in hypothesis generation. This clearly reflects the exploratory nature of the study, and its findings should not be applied to direct clinical decision-making.

## Discussion

ICH occurs due to vessel rupture (2, 18) with hypertension being the most common cause (1, 19, 20). Long-term hypertension induces arteriolar pathological changes (21, 22), making vessels susceptible to rupture during sharp increases in blood pressure. In our study, more than 60% of the ICH patients in both the unmatched cohort and the PSM cohort had a history of hypertension. These findings further demonstrate that hypertensive ICH is the most common type of ICH. Our findings somewhat contrast those of Brouwers et al. (3), but align with Dowlatshahi et al. (23), who reported an HE incidence of 13–32%.The discrepancy observed between our study and Brouwers et al. (3) may be attributed to specific inclusion criteria in this research, such as restricting the time window from onset to baseline imaging to within 24 h, or to differences in population characteristics.

In the acute-phase treatment of ICH, controlling hypertension (24–26) and intracranial pressure (27, 28) is the central objective and is aimed at reducing the risk of cerebral oedema and neurological deterioration (29, 30). Commonly used antihypertensive agents and intracranial pressure-lowering medications, such as thiazide diuretics, mannitol, and furosemide, can all lead to decreased serum potassium levels. Current evidence is insufficient to establish that this drug-induced reduction in serum potassium directly causes HE. HE is associated with coagulation dysfunction (31–33). This study suggests that low serum potassium levels are associated with HE, but whether this affects the coagulation process by increasing vascular permeability (16) requires further investigation. Additionally, studies have indicated a correlation between low serum potassium levels and increased mortality in patients with ICH (34, 35).

In this context, structured acute response protocols such as Code ICH have emerged as essential tools for the timely recognition and management of ICH (see code ICH a call to action) (36). Incorporating laboratory monitoring (including serum electrolyte indicators such as potassium ions) into the Code ICH workflow may increase the precision of acute-phase interventions. This approach focuses on modifiable yet often overlooked factors in the pathophysiological process of ICH, potentially offering further support for improving patient outcomes in the future.

Our study has several limitations. First, our study is a single-center retrospective analysis and may be subject to bias. Second, as a retrospective observational study, key confounding factors that may influence HE—such as the use of acute-phase antihypertensive and intracranial pressure–lowering medications, prior medication history affecting coagulation function, and the CTA spot sign—were not systematically analyzed. Furthermore, serum potassium levels were not examined in further subdivided ranges. Therefore, the association between low serum potassium levels and HE after ICH should be considered a preliminary identification of a potential biomarker. These findings should not be used to guide specific clinical management or correction strategies. Future prospective studies are needed to systematically collect these key data, validate the reliability of these findings, and clarify the value of serum potassium levels more clearly within a multifactorial clinical context.

## Conclusion

In summary, our study demonstrates that low serum potassium levels are associated with HE following ICH, offering a new perspective for understanding the pathophysiological processes of ICH.

## Data Availability

The original contributions presented in the study are included in the article/Supplementary material, further inquiries can be directed to the corresponding authors.
