# Supplementary material for: Association between serum potassium levels and haematoma expansion in intracerebral hemorrhage: a retrospective cohort study
Source: Front Neurol. 2026 Mar 18;17:1707430. doi: 10.3389/fneur.2026.1707430 (PMC13038541; doi:10.3389/fneur.2026.1707430)
Supplement: Supplementary file 1 [file Data_Sheet_1.pdf]

Supplemental Material

Table S1. Comparison of serum potassium levels between the HE and non-HE subgroups on days 1, 3, 7, and 14.

Time	Non-haematoma expansion (n = 32)	Haematoma expansion (n = 28)	*p*-value
D1	3.94 ± 0.48	3.72 ± 0.52	0.177
D3	3.83 ± 0.44	3.75 ± 0.39	0.476
D7	3.88 ± 0.41	3.89 ± 0.54	0.662
D14	4.11 ± 0.54	4.25 ± 0.59	0.578

Notes: Expressed as mean ± standard deviation. HE, haematoma expansion.

Figure S1.

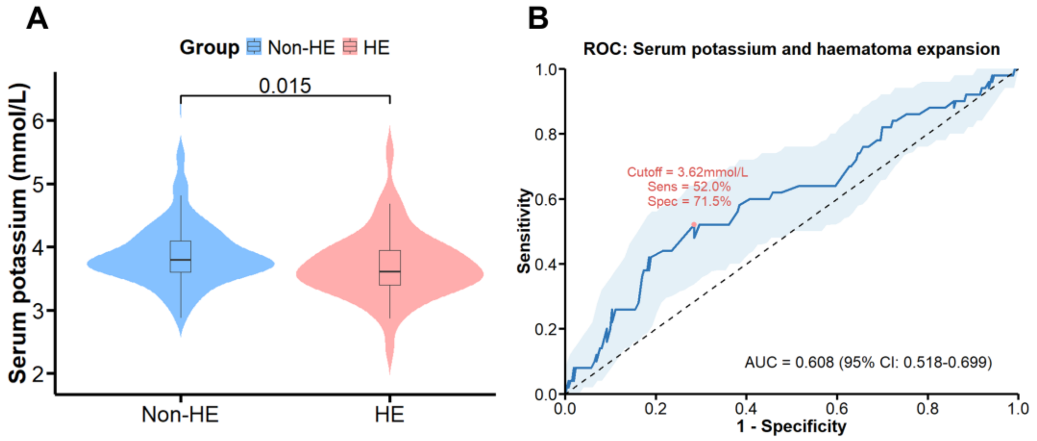

Figure S1. Correlation of low serum potassium levels with HE in ICH patients in an unmatched cohort. (A) Serum potassium levels in the HE subgroup and the non HE subgroup. (B) ROC curve of serum potassium levels for HE. ICH, intracerebral haemorrhage; HE, haematoma expansion; ROC, receiver operating characteristic; AUC, area under the curve.

Figure S2.

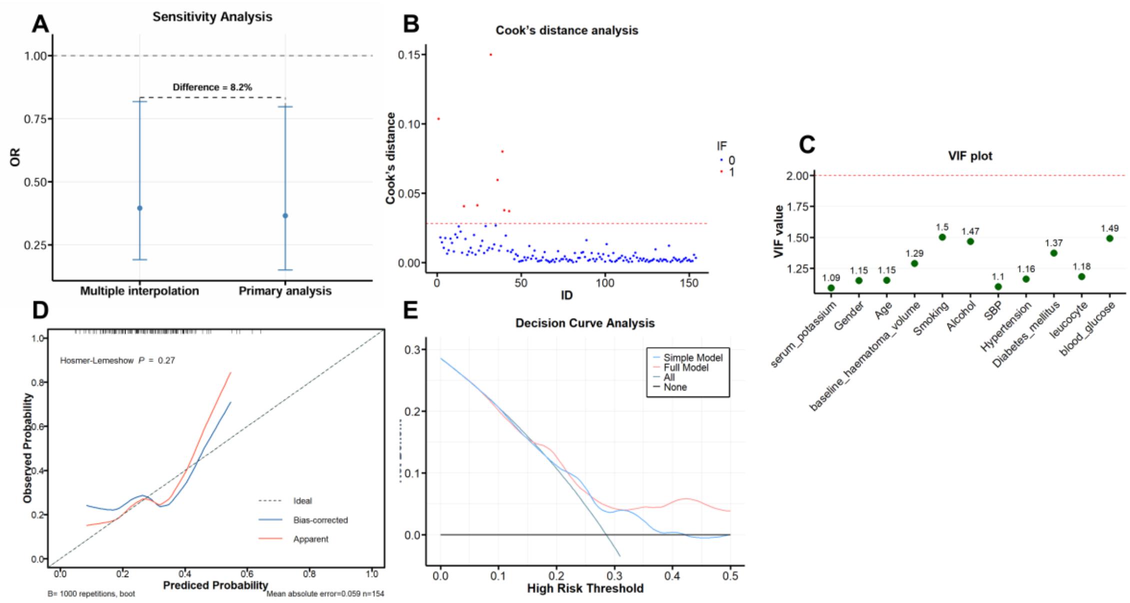

Figure S2. Assessing the robustness of research findings. (A) Multiple interpolation sensitivity analysis evaluates the impact of missing data on model stability. (B) Cook's distance identifies outliers that exert an excessive influence on the model. (C) Calculate VIF to assess multicollinearity among independent variables. (D) Assessing model overfitting using Bootstrap internal validation and the Hosmer-Lemeshow test. (E) Clinical net benefit of DCA evaluation models. VIF, variance inflation factor; DCA, decision curve analysis.

Figure S3.

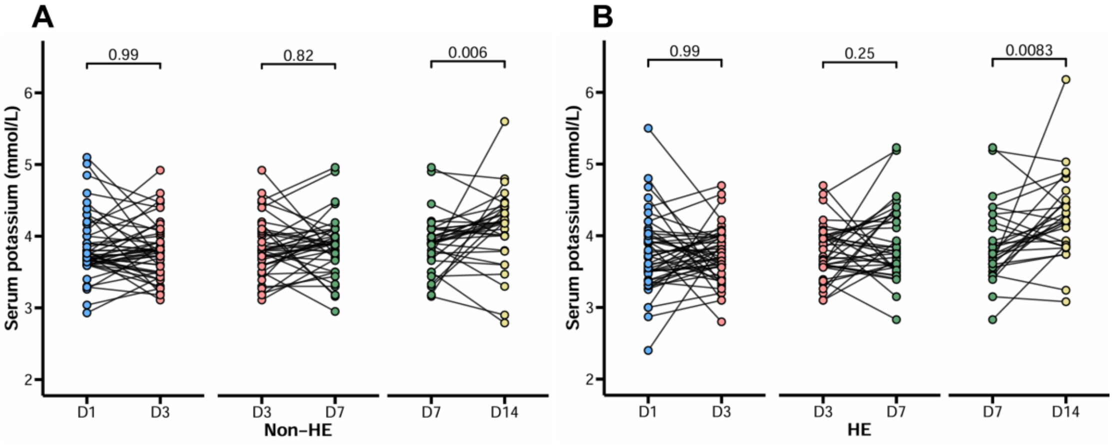

Figure S3. Dynamics of serum potassium levels in patients with ICH. (A) Changes in serum potassium on days 1, 3, 7, and 14 in the non-HE subgroup. (B) Changes in serum potassium on days 1, 3, 7, and 14 in the HE subgroup. ICH, intracerebral haemorrhage; HE, haematoma expansion.
